# Age-Dependent Protective Effect of Selenium against UVA Irradiation in Primary Human Keratinocytes and the Associated DNA Repair Signature

**DOI:** 10.1155/2018/5895439

**Published:** 2018-02-22

**Authors:** C. Favrot, D. Beal, E. Blouin, M. T. Leccia, A. M. Roussel, W. Rachidi

**Affiliations:** ^1^University Grenoble Alpes, 38000 Grenoble, France; ^2^Commissariat à l'énergie atomique et aux énergies alternatives (CEA), Institut Nanosciences et Cryogénie (INAC), Systèmes Moléculaires et NanoMatériaux pour l'Energie et la Santé (SyMMES), Lésions des Acides Nucléiques (LAN), 17 avenue des martyrs, 38054 Grenoble Cedex, France; ^3^Labcatal Pharmaceuticals, Montrouge, France; ^4^Department of Dermatology and Venereology, CHU Grenoble, Grenoble, France

## Abstract

Few studies have focused on the protective role of selenium (Se) against skin aging and photoaging even though selenoproteins are essential for keratinocyte function and skin development. To the best of our knowledge, the impact of Se supplementation on skin cells from elderly and young donors has not been reported. Therefore, the main objective of our study was to evaluate the effects of Se supplementation on skin keratinocytes at baseline and after exposure to ultraviolet A (UVA) irradiation. Low doses of Se (30 nM) were very potently protective against UVA-induced cytotoxicity in young keratinocytes, whereas the protection efficiency of Se in old keratinocytes required higher concentrations (240 nM). Additionally, the DNA repair ability of the old keratinocytes drastically decreased compared with that of the young keratinocytes at baseline and after the UVA exposure. The Se supplementation significantly enhanced the DNA repair of 8-oxoguanine (8oxoG) only in the keratinocytes isolated from young donors. Therefore, aged keratinocytes have an increased vulnerability to oxidative DNA damage, and the Se needs in the elderly should be considered. Strengthening DNA repair activities with Se supplementation may represent a new strategy to combat aging and skin photoaging.

## 1. Introduction

A low dietary selenium (Se) intake increases an organism's susceptibility to oxidative stress-related diseases. Several *in vitro* animal models and human studies have demonstrated an inverse association between dietary Se intake and cancer risk (for review see [1]). Se likely exerts its cancer prevention effects via distinct mechanisms, such as redox regulation, stimulation of apoptosis [2], activation of p53 [3], enhancement of immune functions [4], or induction of DNA repair processes [5]. We recently published a review regarding the chemopreventive activity of Se and proposed potential mechanisms explaining the role of Se in DNA damage repair (for review see [1]). The benefits of Se may result not only from the selenoproteins, which play a very important role in antioxidant defense and maintenance of the cellular reducing environment, but also from the increases in certain DNA glycosylase activities, which are involved in the repair of oxidative DNA damage and certain DNA repair pathways, including those mediated by p53, BRCA1, and Gadd45. The tumor suppressor protein p53 is well known for its apoptotic role in cancer prevention, but p53 also plays an essential role in DNA repair pathways due to its relationship with the APE1 protein, which is an enzyme involved in DNA base excision repair (BER) [3, 6]. Moreover, we recently showed that human prostate-derived LNCaP cells were protected against UVA-induced genotoxicity following a pretreatment with low doses of Se, and Se stimulated the repair of oxidative DNA lesions. This effect was likely due to an enhancement in the activity of OGG1, which is a glycosylase that is responsible for the repair of the major oxidative DNA lesion (7,8-dihydro-8-oxoguanine (8-oxoGua)) [7]. However, these interesting results must be verified in human primary cells to provide more realistic experimental data than those derived from immortalized or transformed cell lines, such as LNCaP. We chose to use primary human keratinocytes, which are the main cells in the epidermis.

Why should we focus on the impact of Se on skin cells?

Se and the selenoproteins are essential for keratinocyte function and skin development. A lack of selenoenzymes in the mouse epidermis leads to abnormalities in the skin and hair follicles, premature skin aging, and premature death [8]. Moreover, the skin, which is the largest body organ, is constantly exposed to oxidative stress, such as UV radiation, chemicals, and pollutants, which may cause skin disorders, including skin cancer [9] and skin aging [10]. Therefore, antioxidants are necessary for optimal skin function. Se dietary supplements or topical applications have been shown to prevent UVB-induced skin lesions and tumors in hairless mice [11]. Additionally, several groups have shown that Se pretreatment can drastically protect keratinocytes, melanocytes, and fibroblasts from UV-induced cytotoxicity (for review see [12]).

UVA is becoming a topic of increasing interest. Currently, UVA is believed to induce DNA damage, such as cyclobutane pyrimidine dimers (CPD) and reactive oxygen species (ROS), which lead to the formation of single-strand breaks (SSB) and oxidized bases, such as 8oxoG, leading to harmful consequences, such as skin aging and carcinogenesis [13]. Moreover, UVA penetrates human skin more effectively than UVB. Because the abundance of UVA is greater than that of UVB, UVA is believed to greatly contribute to skin cancer. This contribution is not due to direct UVA absorption by DNA but rather is due to an indirect mechanism involving ROS. ROS are responsible for the formation of the mutagenic modified base 8oxoGua [14, 15]. This mutation can cause a G to T transversion [16], which is responsible for cancers. Moreover, even if the DNA only weakly absorbs UVA, UVA-induced CPD has been observed in numerous studies investigating cell lines [17] and human skin [18].

Our study aimed to assess the effects of Se supplementation on primary human keratinocytes obtained from normal skin biopsies of two groups of donors, that is, elderly (60–70 years old) and young (20–30 years old) individuals, which could reflect different genetic backgrounds and physiological conditions, at baseline and after exposure to UVA irradiation. Moreover, using our multiplexed DNA repair assay, we assessed the DNA repair signature in primary keratinocytes from different aged donors with or without the Se supplementation. We showed that the low doses of Se (30 nM) very potently protected against UVA-induced cytotoxicity in the young keratinocytes, whereas Se was protective in the old keratinocytes only at higher concentrations (240 nM). In addition, we found that the DNA repair capacities of the old keratinocytes were drastically lower than those of the young keratinocytes. The supplementation with Se significantly enhanced the global DNA repair capacities, particularly in cells isolated from young donors.

## 2. Materials and Methods

### 2.1. Skin Sample Preparation and Cell Culture

The human epidermal keratinocyte cultures were established by outgrowth from biopsies obtained immediately after breast plastic surgery from healthy donors with their informed consent (Centre Hospitalier de Grenoble, Grenoble, France). The donors were 20–30 or 60–70 years old. All donors were Caucasian and phototype II or III. The biopsies were preserved in DMEM-PS (medium containing 100 units/mL penicillin and 100 *μ*g/mL streptomycin) and kept at 4°C until the keratinocyte extraction. Then, the biopsies were immersed overnight in 0.25% trypsin at 4°C. Subsequently, the dermis and epidermis were separated using a pair of forceps, and the epidermis was incubated for 60 min at room temperature with PBS containing 0.125% trypsin and 0.05% EDTA with magnetic stirring. After adding 10% FCS, the cells were resuspended and filtered through a 70 *μ*m cell strainer (Becton–Dickinson) to remove the remaining aggregates before counting. After the extraction, cells were cultured in keratinocyte serum-free medium (KSFM) supplemented with 1.5 ng/mL EGF, 25 *μ*g/mL bovine pituitary extract (BPE), and 75 *μ*g/mL of primocin in culture flasks at 37°C and 5% CO_2_. For all experiments, the cells were used at passages 2 or 3.

For the Se treatment, the media was supplemented with either 30 nM or 240 nM of sodium selenite (SS) for 72 h. A no treatment (NT) control was included in each experiment.

### 2.2. UVA Irradiation and Cytotoxicity Assay

The keratinocytes were plated in 35 mm plates and incubated at 37°C for 72 h in the presence of 30 or 240 nM of Se. Then, the culture medium was removed, the cells were rinsed twice with PBS, and irradiation with UVA (UVA 700L Waldmann, Germany) was performed at different doses (0, 25, 50, and 100 J/cm^2^) in PBS with the lead removed. The plates were placed to ice to prevent heating due to the UVA lamp. Finally, the cell viability was determined using the MTT assay 24 h after the UVA irradiation as previously described [19].

### 2.3. Multiplexed Enzymatic DNA Repair Assay Using a Biochip

#### 2.3.1. Preparation of Whole Protein Extracts

Thawed cells were washed twice with ice-cold PBS. Then, the pellets were suspended in 1 mL of ice-cold buffer A (10 mM HEPES pH 7.9, 1.5 mM MgCl_2_, 10 mM KCl, 0.01% Triton X-100, 0.5 mM DTT, and 0.5 mM phenylmethanesulfonyl fluoride (PMSF)). After a 20 min incubation on ice, the lysates were cleared by 5 min centrifugation at 13,000 rpm at 4°C and stored frozen in 100 *μ*L aliquots at −80°C. The protein concentration in each sample was determined using the BCA Kit (Interchim, Montluçon, France).

#### 2.3.2. Multiplexed Cleavage Assay

We used a previously described [20, 21] multiplexed ODN array, which consists of ODNs containing different base lesions that are hybridized to one complementary ODN at specific locations on a 24-well glass slide (the multiplexed ODN principle is described in Supplemental Figure S1). Biotinylated support ODNs were printed on streptavidin glass slides (Xantec bioanalytics GmbH, Germany) in duplicate in a 24-well format. The wells were subsequently individualized by setting the slides into ArrayIt® microplate hardware. Duplexes preformed through the specific hybridization of one Cy3-labeled lesion ODN and one long ODN were hybridized on support ODNs for 1 h at 37°C in a total volume of 80 *μ*L. Each long ODN has a part complementary to a lesion ODN and a part complementary to a specific support ODN. This latter part directs the hybridization onto a specific location through the support ODN. Slides were then rinsed three times for 5 min with 80 *μ*L of excision buffer (10 mM HEPES/KOH, pH 7.8, 80 mM KCl, 1 mM EGTA, 0.1 mM ZnCl2, 1 mM DTT, and 0.5 mg/mL BSA).

Each well contained a control ODN (lesion-free ODN) and eight lesion-containing ODNs that were available in duplicate as follows: 8oxoG paired with cytosine (8-oxoG-C); adenine paired with 8oxoG (A-8oxoG); thymine glycol paired with adenine (Tg-A); tetrahydrofuran, which is an AP site substrate equivalent, paired with adenine (THF-A); hypoxanthine paired with thymine (Hx-T); ethenoadenine (EthA) paired with thymine (EthA-T); and uracil paired with adenine (U-A) or guanine (U-G). The lesions were labeled with a Cy3 fluorophore at their ends. The total protein extracts were added to the wells, and the excision rate of each lesion by the enzyme contained in each extract was quantified by measuring the fluorescence loss at each site. This outcome was calculated as a percentage, and the fluorescence level of the control (which corresponded to the well incubated only with the excision buffer) served as a reference (100%).

The excision reactions were conducted using a protein concentration of 20 *μ*g/mL at 30°C for 30 min in 80 *μ*L of the excision buffer (10 mM HEPES pH 7.9, 80 mM KCl, 1 mM EGTA, 0.1 mM ZnCl_2_, 1 mM DTT, and 0.5 mg/mL BSA). The excision reaction was stopped by washing the slides 3 times for 5 min with the washing buffer (PBS/0.2 M NaCl and 0.1% Tween 20). The fluorescence of each spot was quantified at 532 nm using a Genepix 4200A scanner (Axon Instrument, Molecular Devices, Sunnyvale, CA, USA) and the Genepix Pro 5.1 software (Axon Instrument). The results of replicates (4 spots) were normalized using the Normalizelt software as described by Millau et al. [22].

## 3. Results

### 3.1. Protective Effect of Se on UVA-Induced Cytotoxicity and the Impact of Age

**Figure 1:**
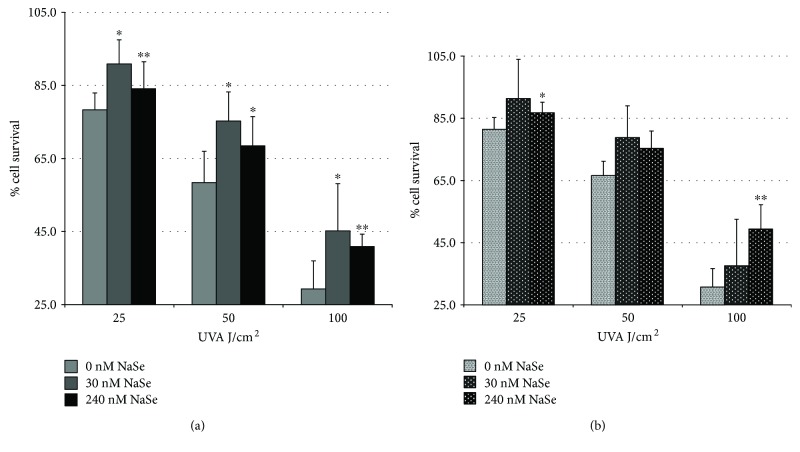
SS age dependently increases resistance to UVA. Keratinocytes obtained from (a) young donors or (b) elderly donors were pretreated with 30 nM or 240 nM of SS. Then, the keratinocyte viability was determined using an MTT assay 24 h after exposure to increasing doses of UVA. Values are expressed as the mean ± SD of (*n* = 3 donors, 4 independent measurements for each age group), and we tested for statistical significance using Student's *t*-test. ^∗^ *p* < 0.05 and ^∗∗^ *p* < 0.01 versus nontreated (nM SS) keratinocytes.

Figures 1(a) and 1(b) show the % viability of the primary keratinocytes obtained from young and elderly donors that were irradiated with increasing doses of UVA (25, 50, and 100 J/cm^2^) after pretreatment with 30 nM or 240 nM of SS for 72 h. Overall, regardless of the level of the Se supplementation, the keratinocytes that were pretreated with Se exhibited better survival than the NT controls after the UVA irradiation. However, the protective effect of Se against UVA varied according to the age of the donors, and Se more effectively protected the keratinocytes obtained from the young donors. Note that the pretreatment of control nonirradiated young (Supplemental Figure S2A) and old (Supplemental Figure S2B) keratinocytes with 30 or 240 nM of Se does not have any significant effect on cell proliferation or cell viability.

After the exposure to 100 J/cm^2^ of UVA, the pretreatment of the young keratinocytes with 30 nM or 240 nM Se significantly increased the cell viability from 29% (in the NT cells) to 45% (*p* < 0.05, in the young keratinocytes pretreated with 30 nM Se versus NT) or 41% (*p* < 0.01, in the young keratinocytes pretreated with 240 nM Se versus NT) (Figure 1(a)). After the exposure to 100 J/cm^2^ UVA, only the pretreatment of the old keratinocytes with 240 nM of Se significantly increased cell survival from 30% (in the NT cells) to 50% (*p* < 0.01, in the old keratinocytes pretreated with 240 nM Se versus NT) (Figure 1).

Low doses of Se (30 nM) might very potently protect against UVA-induced cytotoxicity in young keratinocytes, whereas the protection efficacy of Se in old keratinocytes was only observed at higher concentrations (240 nM). Thus, aged keratinocytes require four times the amount of SS (240 nM) than that required by young keratinocytes (30 nM) to be protected from UVA-induced cytotoxicity.

### 3.2. Impact of Aging on DNA BER Activities in Keratinocytes and the Effect of Se Supplementation

#### 3.2.1. Effect of Aging

**Figure 2:**
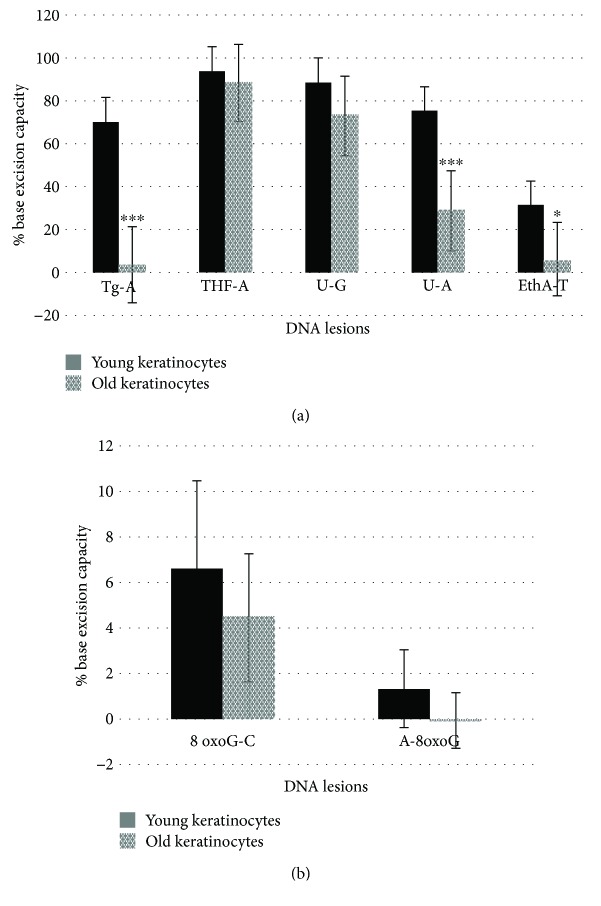
Impact of aging on DNA BER capacities of keratinocytes. Using the ODN biochip, cellular extracts of young or old keratinocytes were tested for their excision activities for (a) Tg-A, THF-A, U-G, U-A, and EthA-T and (b) 8oxoG-C and A-8oxoG. Values representing the excision capacity are expressed as the mean percentage of cleavage of total fluorescence intensity ± SD with respect to the initial fluorescence intensity (*n* = 2 donors, 3 independent measurements *n* = 3 for each age group). ^∗^Significantly different (*p* < 0.05), ^∗^ *p* < 0.005, and ^∗∗∗^ *p* < 0.0005.

To obtain additional information regarding the DNA repair activities in the keratinocytes obtained from the two age groups and evaluate the effect of the Se supplementation, we used our newly developed multiplexed ODN cleavage assay, which allows for the quantification of the excision capacity of several glycosylases associated with the BER pathway. As previously shown by our laboratory using the same ODN cleavage assay [23], the excision percentages were high (≥70%) for the Tg-A, THF, U-G, and U-A lesions with cell extracts (Figure 2(a)), intermediate for the EthA-T lesion (≤30%) (Figure 2(a)) and very low for the 8-oxoG-C and A-8oxoG lesions (less than 5%) (Figure 2(b)). Overall, a drastic decrease in the excision capacity of Tg-A (*p* < 0.001), U-A (*p* < 0.01), and EthA-T (*p* < 0.05) was observed in the keratinocytes obtained from the elderly donors compared with that in the keratinocytes obtained from the young donors. The excision capacities of Tg-A, U-A, and EthA-T were decreased by 19.4, 2.5, and 5.2, respectively, in the old keratinocytes compared with those in the young keratinocytes (Figure 2(a)). However, no significant effect was observed for the other lesions such as 8-oxoG-C and A-8oxoG lesions (Figure 2(b)).

#### 3.2.2. Effect of Se Supplementation

To study the effect of the Se supplementation on the DNA repair capacity, we used the same multiplexed ODN biochip to measure the excision capacity of the DNA lesions by protein extracts from keratinocytes pretreated with 240 nM of Se for 72 h. The induction or inhibition of the DNA repair activity due to the Se supplementation was evaluated by calculating the ratio of the excision capacity of each lesion with Se to the excision capacity of each lesion without Se in each cell type (i.e., young or old keratinocytes).

**Figure 3:**
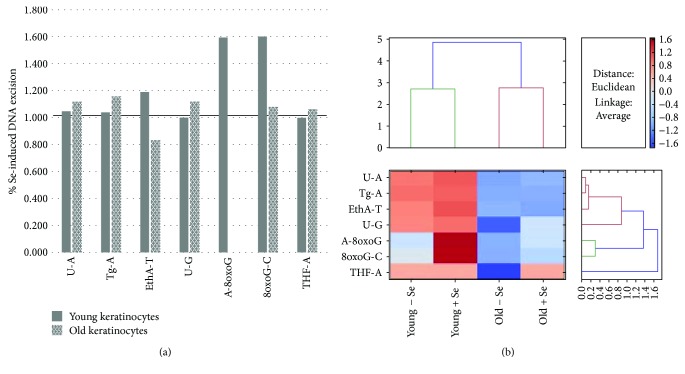
The effect of Se supplementation on the DNA repair capacity. Cellular extracts of young or aged keratinocytes were tested for their excision capacity using the ODN biochip. The mean percentages of the excision capacity (*n* = 2 donors, 3 independent measurements *n* = 3 for each age group) were calculated, and the mean ratios of the Se-treated/NT control for each young or old keratinocyte sample were calculated for each lesion (a). A heatmap representation of the variation in the excision activities in the nonsupplemented and supplemented young and old keratinocytes is shown in (b).

For the keratinocytes obtained from the young donors, the excision capacities of 8-oxoG-C and A-8oxoG were enhanced by 1.6- and 1.59-fold in the Se-treated cells, respectively. However, no significant enhancement was observed in other lesions after the Se pretreatment (Figure 3(a)).

For the keratinocytes obtained from the elderly donors, no significant effects were observed on the DNA excision capacity after the Se pretreatment (Figure 3(a)).

Finally, a heatmap of the variation in the excision activities in the nonsupplemented and supplemented young and old keratinocytes is shown in (Figure 3(b)) which represents the absolute variation in the excision activities (Euclidean dissimilarity measure).

## 4. Discussion

Recently, pretreatment with low doses of SS (30 nM for 72 h) has been shown to sufficiently provide protection to LNCaP cancer prostate cells against UVA irradiation. Moreover, Se-treated LNCaP cells exhibited an increased oxidative DNA repair capacity [7]. Therefore, the benefits of Se could be due to a combination of enhancing antioxidant defenses through the selenoproteins, preventing DNA damage and increasing the DNA repair capacities. However, these interesting results needed to be verified in nontumorigenic or nontransformed cell lines to better approximate normal physiological conditions. The skin is one of the most exposed organs to environmental insults, such as UV, and the selenoproteins are essential for keratinocyte function and skin homeostasis because the specific suppression of the selenoproteins in epidermal cells results in several skin abnormalities and alopecia in knockout mice [8]. In our study, we used primary human keratinocytes obtained from normal skin biopsies of elderly and young donors. Investigating primary human keratinocytes from various donors of different ages allowed us to confirm whether SS is active despite the differences in the donors' genetic background, polymorphisms, or physiological conditions. Very recently, we used the same biological materials (keratinocytes obtained from old (60–70 years old) and young donors (20–30 years old), in order to study the proteomic signature of these skin cells. A total of 517 unique proteins were identified, and 58 proteins were significantly differentially expressed with 40 that were downregulated and 18 upregulated in keratinocytes obtained from old donors when compared to young ones. Gene ontology and pathway analysis performed on these 58 putative biomarkers of skin aging evidenced that several important “aging pathways” were modulated in old keratinocytes (inflammation, oxidative stress, cytoskeleton…) [24].

First of all and in order to select the optimal concentration of SS to be included in our culture condition, we showed that SS is well tolerated by keratinocytes below 500 nM concentrations. This result is consistent with several studies showing that there is no toxicity observed in several primary or transformed cell lines treated with a range of SS concentrations below 500 nM [25, 26]. However, higher concentrations of SS showed high toxicity in several cell lines (e.g., IC50 of sodium selenite 2.3 *μ*M on HaCat cell line). It has been shown that high concentrations of SS could induce cell death by their ability to induce apoptosis via ROS production. This may directly imply the reduced form of selenite, the selenide, which is able to form ROS via the FAD-containing enzyme thioredoxin reductase (TR). This reaction is considered able to transform Se from antioxidants to prooxidants [27].

Using an MTT cell survival assay, we showed that the low doses of Se (30 nM of SS) were very potently protective against UVA-induced cytotoxicity in the young keratinocytes, whereas the protection efficacy of Se in the old keratinocytes was observed only with the higher Se concentrations (240 nM of SS). This difference in the effectiveness of protection by Se could be explained by the higher uptake of Se by the young keratinocytes than by the old keratinocytes (data not shown). This hypothesis is consistent with several epidemiological studies that showed a general decrease in the plasma Se concentrations with age in healthy elderly individuals [28]. Aging-related decrease in the plasma Se levels could weaken the antioxidant defenses and enhance susceptibility to several degenerative diseases, such as cancer, cardiovascular diseases, or cognitive decline [29]. In contrast, the plasma Se level is a good predictor of longevity in the elderly population [30–32].

We have previously shown that Se supplementation may exert its benefits by enhancing the DNA damage repair activity, and in this study, we examined the DNA repair capacities of young and old keratinocytes at baseline and after Se pretreatment (240 nM SS) using a multiplexed DNA biochip recently developed in our laboratory. At baseline, a drastic decrease in the excision capacity of several base lesions was observed in the old keratinocyte extracts compared with that in the young keratinocyte extracts. A drastic effect of aging was observed on the excision of the Tg lesion. Tg is one of the major toxic oxidative DNA lesions generated by ROS [33] and possesses strong blocking properties for replication and transcription, which leads to cell death [34]. Tg lesions in mammalian cells are excised by the DNA glycosylase endonuclease III-like protein 1 (Nth1) [35]. In consistent with our data, a significant decrease in the excision efficiency of Tg in protein extracts from old fibroblasts has been shown [23]. Furthermore, Nth1 deficiency and an accumulation of Tg lesions have been recently shown to enhance telomere fragility in mice and contribute to cellular aging [36]. Moreover, in contrast to the excision of U-G, which is not affected by aging, the repair of U-A significantly declines in old keratinocytes. Although the misincorporation of uracil opposite A is believed to be nonmutagenic, it may modulate DNA transcription by interfering with the sequence-specific binding of transcription factors, such as AP-1 [37]. Different -omic studies investigating aged skin have shown dramatic modulations in the expression levels of several genes and/or proteins involved in proliferation, differentiation, inflammation, and the regulation of transcription [38]. In humans, four distinct uracil–DNA glycosylases (i.e., UNG2, SMUG1, TDG, and MBD4) are involved in the removal of uracil that has been misincorporated into DNA. The most well-known uracil–DNA glycosylase is UNG2, which plays a key role in the initiation of the excision of misincorporated uracil [39]. Interestingly, the suppression of UNG reduces cell proliferation, induces apoptosis, and increases the cellular sensitivity to genotoxic stress [40]. Finally, the excision repair of EthA was also decreased in the old keratinocytes. EthA is a lipid peroxidation- (LPO-) base adduct that is generated by the exposure of DNA to lipid peroxides, such as 4-hydroxy-2-nonenal (HNE) or 4-oxo-2-nonenal. Etheno adducts have strong mutagenic and carcinogenic potentials and play a major role in aging and aging-related diseases [41]. EthA is excised from DNA by alkylpurine *N*-DNA glycosylase (ANPG) [42], which is a monofunctional glycosylase that requires an AP endonuclease to continue the BER process. In summary, the aging of keratinocytes could act as a double-edged sword on DNA integrity by increasing the accumulation of lesions in the genome via oxidative stress products (such as LPO products) and decreasing DNA repair capacities.

SS was previously demonstrated to have the capacity to improve DNA repair by increasing antioxidant enzymes, such as GPx1 or TrxR, which are involved in pathways involving other proteins known for their anticancer properties, such as APE1 and p53 [43]. Seo et al. demonstrated that the SS concentration is a determinant of p53 activity, and protection from DNA damage by SS compounds is p53-dependent [5, 44]. These authors state that selenomethionine, which is the organic form of SS, can activate p53. This activation requires the redox factor APEX1 through a redox mechanism independent of DNA damage. The involvement of p53 in the BER pathway has been highlighted by Offer et al. and Zhou et al. [45–47] Consistently, p53 is able to enhance the combined activities of OGG1 and APEX1 to remove 8oxoG lesions [6]. Therefore, Se plays an important role in the modulation of DNA repair capacities in tumorigenic cell lines and primary fibroblasts despite the physiological differences in these systems. Thus, we measured the DNA excision capacities after Se pretreatment (240 nM of SS) in young and old keratinocytes and showed that the supplementation with Se specifically enhanced the DNA repair machinery of the 8oxoG lesions in the young keratinocytes only. Indeed, protein extracts from young keratinocytes pretreated with Se displayed enhanced cleavage of 8oxoG-C and A-8oxoG. 8oxoG is a major mutagenic purine lesion caused by ROS attacks on DNA. 8oxoG is primarily removed from 8oxoG-C pairs by OGG1. When 8oxoG escapes this repair and subsequent replication occurs, the polymerases incorporate an A across the 8oxoG, leading to an A-8oxoG mispair. In mammalian cells, MYH excises adenine bases that are misincorporated opposite to 8oxoG and recruits AP endonuclease to cleave the residual AP sites [48]. Hence, MYH serves as a backup system for the removal of 8oxoG. The improved excision capacity of 8oxoG in young keratinocytes treated with Se may be due to the ability of Se to maintain a reduced cellular environment because several DNA repair enzymes are sensitive to the redox status of cells. For example, OGG1 is a redox-sensitive enzyme, and its activity can be completely inhibited after prooxidant treatment [49]. In addition, selenium may be impacting the activity of glycosylases by altering posttranslational modifications. Indeed, several glycosylases are regulated by acetylation and/or phosphorylation and selenium has been shown to alter histone deacetylase and kinase activities. Moreover, selenium has been shown to alter promoter DNA demethylation [50] and may modulate the expression of BER-associated gene. Finally, in contrast to the results obtained in the young keratinocytes, the supplementation with Se had no effect on the repair activities in the aged keratinocytes regardless of the type of lesion. This lack of response is mysterious and could be due to irreversible decline in multiple physiological functions and metabolic pathways in aged keratinocytes, making them unable to respond to their environment. Indeed, several transcriptomic studies have investigated the effect of aging on gene expression in several model organisms and humans and have shown that differentially expressed genes in elderly and young human male skin were involved in various cellular processes, such as metabolism, signal transduction, apoptosis, and the regulation of transcription [38].

In summary, to the best of our knowledge, our data are the first to show that aged keratinocytes require four times more SS (240 nM) than young keratinocytes (30 nM) to be protected from UVA-induced cytotoxicity. We also demonstrated that old keratinocytes have a drastically lower DNA repair capacity than young keratinocytes. Moreover, the Se-treated young keratinocytes exhibited increased repair activities of 8oxoG, which indicates a new genoprotective property of Se against the major mutagenic purine lesion. However, no effect of the Se treatment was observed on the DNA repair activity in the old keratinocytes. Overall, these original data strongly suggest an increased vulnerability of keratinocytes with age, and the Se needs in the elderly should be considered. Future studies should help us better understand the regulation of DNA repair by Se and the effects of aging.

## 5. Conclusion

Few studies have focused on the protective role of Se on skin aging and photoaging even though the selenoproteins are essential for keratinocyte function and skin development. Our aim was to evaluate the effects of Se supplementation on primary skin keratinocytes obtained from normal biopsies of elderly and young donors at baseline and after exposure to UVA irradiation. Low doses of Se were very potently protective against UVA-induced cytotoxicity in young keratinocytes, whereas the aged keratinocytes require four times more Se than the young keratinocytes to be protected from UVA-induced cytotoxicity. Moreover, we showed that the old keratinocytes had a drastically lower DNA repair capacity than the young keratinocytes at baseline, and the Se supplementation only significantly enhances the DNA repair of 8oxoG in keratinocytes isolated from young donors. These original data strongly suggest an increased vulnerability of aged keratinocytes to oxidative damage, and the Se needs in the elderly should be considered. Strengthening DNA repair activities with Se may represent a new strategy to combat aging and skin photoaging. These results highlight the protective mechanism of Se and, therefore, could be used to identify new targets for UVA exposure protection.

## Supplementary Materials

Supplementary Materials Supplemental Figure 1: the ODN biochip principles. (A) Biotinylated support ODNs are spotted on streptavidin glass slide through a piezo dispense capillary. At that point, no fluorescence can be recorded after scanner reading, but the localization of the spots can be discerned (blue circles). (B) A first step of hybridization of the long and lesion ODNs is performed for 1 h at 37°C in vitro, to form the long/lesion ODN complex. The long/lesion ODN complex is hybridized to the support ODN during a second step of hybridization on the slide. The fluorescence for each spot is then maximal. (C) Finally, the excision reaction is performed by incubating nuclear extracts on the slides. The residual fluorescence is quantified and proportional to the excision capacity of the nuclear extract. Supplemental Figure 2: influence of SS on cell survival. Effects on cell survival after application of a range concentrations of SS during 72 h for keratinocytes obtained from (S2A) young donor or (S2B) elderly donor. Cell survival was determined by the MTT assay, and data were presented as relative to nontreated (0 nM) control cells (*n* = 3 independent measurements for each group age).
