# Supplementary material for: Age-Dependent Protective Effect of Selenium against UVA Irradiation in Primary Human Keratinocytes and the Associated DNA Repair Signature
Source: Oxid Med Cell Longev. 2018 Feb 22;2018:5895439. doi: 10.1155/2018/5895439 (PMC5842700; doi:10.1155/2018/5895439)

**Supplemental Figure 1. The ODN Biochip: principles.** (A) Biotinylated Support ODNs are spotted on streptavidin glass slide, through a piezo dispense capillary. At that point, no fluorescence can be recorded after scanner reading, but the localization of the spots can be discerned (blue circles). (B) A first step of hybridization of the long and lesion ODNs is performed for 1h at 37°C in vitro, to form the long/lesion ODN complex. The long/lesion ODN complex is hybridized to the support ODN during a second step of hybridization on the slide. The fluorescence for each spot is then maximal. (C) Finally, the excision reaction is performed by incubating nuclear extracts on the slides. The residual fluorescence is quantified and proportional to the excision capacity of the nuclear extract.

## Supplemental Figure S1

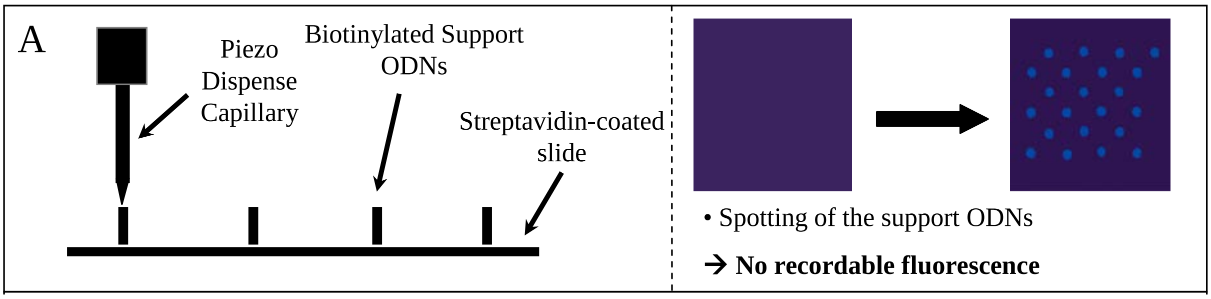

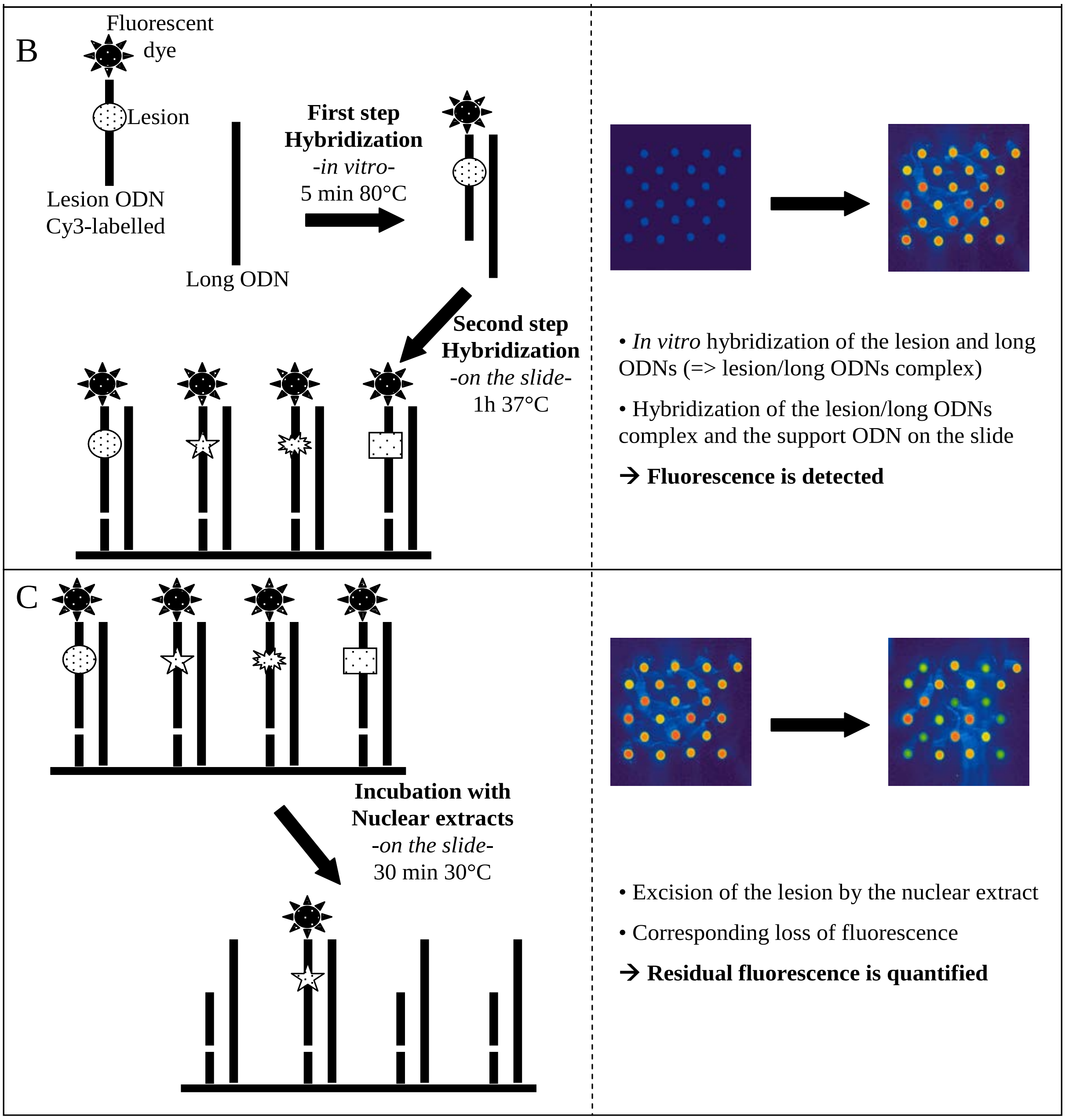
B
Fluorescent dye
Lesion
Lesion ODN
Cy3-labelled
Long ODN
First step
Hybridization
-in vitro-
5 min 80°C
Second step
Hybridization
-on the slide-
1h 37°C
• In vitro hybridization of the lesion and long ODNs (=> lesion/long ODNs complex)
• Hybridization of the lesion/long ODNs complex and the support ODN on the slide
→ Fluorescence is detected
C
Incubation with
Nuclear extracts
-on the slide-
30 min 30°C
• Excision of the lesion by the nuclear extract
• Corresponding loss of fluorescence
→ Residual fluorescence is quantified

**Supplemental Figure 2. Influence of SS on cell survival**. Effects on cell survival after application of a range concentrations of SS during 72h for Keratinocytes obtained from (S2A) young donor or (S2B) elderly donor. Cell survival was determined by the MTT assay and data were presented as relative to non-treated (0 nM) control cells (n = 3 independent measurements for each group age).

**Supplemental Figure S2**

**S2A**

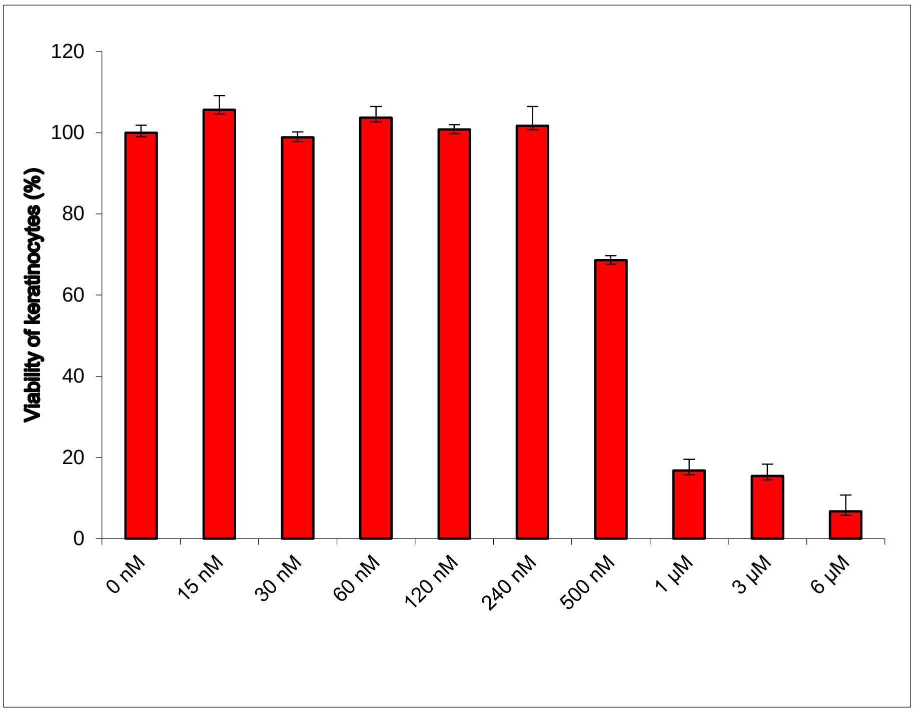


**S2B**

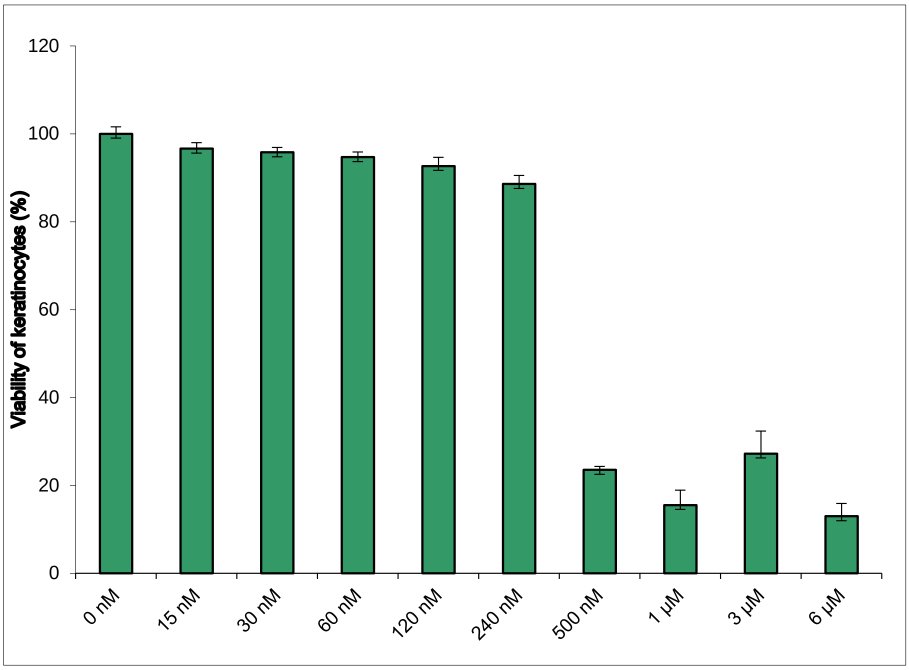

Supplement: Supplementary Materials — Supplemental Figure 1: the ODN biochip principles. (A) Biotinylated support ODNs are spotted on streptavidin glass slide through a piezo dispense capillary. At that point, no fluorescence can be recorded after scanner reading, but the localization of the spots can be discerned (blue circles). (B) A first step of hybridization of the long and lesion ODNs is performed for 1 h at 37°C in vitro, to form the long/lesion ODN complex. The long/lesion ODN complex is hybridized to the support ODN during a second step of hybridization on the slide. The fluorescence for each spot is then maximal. (C) Finally, the excision reaction is performed by incubating nuclear extracts on the slides. The residual fluorescence is quantified and proportional to the excision capacity of the nuclear extract. Supplemental Figure 2: influence of SS on cell survival. Effects on cell survival after application of a range concentrations of SS during 72 h for keratinocytes obtained from (S2A) young donor or (S2B) elderly donor. Cell survival was determined by the MTT assay, and data were presented as relative to nontreated (0 nM) control cells (n = 3 independent measurements for each group age). [file 5895439.f1.pdf]
